# Ensuring Elderly Well-Being During COVID-19 by Using the Internet of Things

**DOI:** 10.1017/dmp.2020.390

**Published:** 2020-10-22

**Authors:** Prateek Pandey, Ratnesh Litoriya

**Affiliations:** JAYPEE University of Engineering and Technology, Raghogarh, Guna, India

**Keywords:** behavior monitoring, COVID-19, disaster management, IoT, vulnerable population care

## Abstract

Coronavirus disease (COVID-19) cases continue to surge, and the world must learn from this disaster. Most of the world economies are shattered due to this pandemic. The development of infrastructure to counter such deadly viral attacks in the future is the wisest investment that can be made. The elderly population is the most vulnerable age group affected by the pandemic, and the threat to their lives becomes manifold if they are living alone. Thus, a well-formed elderly support framework is required to safeguard this vulnerable population from COVID-like disasters in the future. We report here on the research findings we conducted by laying out a mitigation system for elderly well-being during disastrous times. The proposed system demands a sound collaboration between software, hardware devices, the state, and social agencies.

As per the World Health Organization (WHO),^1^ coronavirus disease (COVID-19) cases continue to surge, and the world must learn from this disaster. Most of the world economies are shattered due to this pandemic. The development of infrastructure to counter such deadly viral attacks in the future is the wisest investment that can be made. Another report from the WHO^2^ indicates that the elderly population is the most vulnerable age group affected by the pandemic, and the threat to their lives becomes manifold if they are living alone. Thus, a well-formed elderly support framework is required to safeguard this vulnerable population from COVID-like disasters in the future. We report here on the research findings we conducted by laying out a mitigation system for elderly well-being during disastrous times. The proposed system demands a sound collaboration between software, hardware devices, the state, and social agencies. Due to lockdown around the country and, therefore, the unavailability of the university’s resources, the presented outcomes are based on a pilot study conducted on only 1 elderly subject; however, the authors are determined to continue their research once life returns to “normal.”

We asked elderly subjects (69 years of age) to push a button on a custom-made mobile app when needing to buy important items from the market. The app was specially designed for keeping the cognitive limitations of older adults in mind. On pressing the button, an appropriate message would be sent to a cloud-based service, and the nearest lockdown volunteer was informed through the app and social-media services installed on his/her mobile. This simple but effective socio-technical system helped maintain social distancing norms and the well-being of the elderly subject in trying times. As a consequence of using technologies such as artificial intelligence (AI) and the Internet of things (IoT) in elderly care, the longevity of people can be increased.^3-5^

The IoT concept can be understood as an ecosystem of interconnected computing devices, machines, objects, or people equipped with unique identifiers. It has the data transmission ability over a network without entailing human-to-human or human-to-computer interaction.^6^

AI is a wide-ranging branch of computer science that refers to the simulation of human intelligence in machines that are programmed to think like humans and imitate their actions.^7^ AI is an interdisciplinary technology that incorporates multiple approaches for decision making.^8-10^ When it comes to combining IoT and AI in different applications, including disaster prevention and elderly care, chances are together, and they will perk up operational efficiency in this field.^11^ Soe Ye Yint Tun et al.^12^ provide a wide-ranging overview of IoT and wearable technologies’ applications, including the data type collected and the types of devices for elderly health care. Elena et al.^13^ presented an IoT-based framework aiming at developing smart devices to support older adults living alone. Most of the similar solutions are reactive and require some kind of wearable gadgets to be worn by the elderly all the time or surveillance cameras to be installed around their personal space.

We used this technology a bit differently in that we installed 3 motion sensors in the subject’s house, which tracked the movement of the individual, using an android app installed on the subject’s mobile phone. As a result, the subject did not have to wear any gadgets on the wrist or around the neck, and also there was no need for video surveillance, which can be a violation of their privacy rights. As a backup plan, the IoT-based system sends an “OK” signal after a regular interval. If the system somehow fails or breaks, then the receiver system doesn’t receive the “OK” signal from the subject’s accommodation, and the volunteers are informed to contact the subject to assure its well-being and to fix the broken system.

Actions of the elderly around the day are recorded for further analysis. We defined “W_f_,” the well-being factor, as a function of the movements an elderly individual performed in his house. A move is considered a signal of well-being, in general; however, if an activity is not found in the expected time frame, this doesn’t necessarily mean that the subject is in trouble. The subject’s health credentials and medication he is on play an essential role in decision making. Such health credentials were recorded on a locally made blockchain network for this research project.^14^

Blockchain technology facilitates distributed public ledgers that hold immutable data in an encrypted and secure way.^15^ Here, the block is made up of digital pieces of information shared across a network of computers. Records are immutable, and once a record has been added to the chain, it is tough to change. Blockchains have been used to securely circulate virtual currencies like bitcoin, but many other possible use-cases are emerging.^16-18^

The information available on the blockchain has 2 fields of interest: disease and behavior. Thus, the well-being factor of an elderly is defined as *W*_*f*_ = *f*(*Ø*_*A*_, *θ*_*H*_), where *Ø*_*A*_ represents the statement of activeness relative to the general activity shown by the elderly in the past few days, *θ*_*H*_ being the statement of the health of the elderly, which is acquired from the electronic health records available on a blockchain.

If the subject does not show activity in the expected time frame (say between 4:00 AM and 5:30 AM) but is currently on anti-depressant medication with “drowsiness,” an extended 30 minutes margin can safely be added on both sides of the time frame. In another case, if the subject doesn’t show any movement and is on a diuretic medication (indicated by frequent use of the washroom facility), this is considered an alarming situation, and the social volunteers are informed through various means. In such cases, the expected time frame must be contracted (say about 30 minutes) from both sides. For demonstration purposes, the 8 time frames of 3 hours each are created and presented in Table 1.

**TABLE 1:** Time Frame Activity Demonstration

#	Time Frame	No. of Activities (Last 10 Days)	Average no. of Times the Subject Is Active in the Given Time Frame	No. of Times the Subject Is Active Today	Trend
1	00:00-03:00	3	0	0	=
2	03:00-06:00	7	1	2	+
3	06:00-09:00	115	11	9	-
4	09:00-12:00	142	14	11	-
5	12:00-15:00	70	7	8	+
6	15:00-18:00	65	6	4	-
7	18:00-21:00	89	9	9	=
8	21:00-00:00	45	4	3	-
	**Total**	**536**	**52**	**46**	**-**

**Activities include the aggregated data collected from all the 3 sensors with respect to a particular time frame.

***Motion sensors are installed near the washroom facility, TV room, and kitchen.

Table 1 demonstrates that the subject today is about 11.5% less active. Whether the decline in activity by 11.5% is a cause for worry or just a usual variation is determined according to the national health policies. The same can be recorded in a policy file (policy.json) in the proposed system. Other parameters to be included in a policy file are the min_act_dif_bel, which implies the minimum activity difference threshold below which, if the subject shows inactivity, the concerns will be addressed. Similarly, min_act_dif_abo is the threshold above which, if the subject shows activity, the concerns will be addressed. If the min_act_dif_bel property is set to 3, then on the 11th day, the volunteers will be called for the inactivity in the third time frame, but the same will not be called for the fourth time frame. While setting the limits to these properties, the age and the health status of the subject should also be considered.

Figure 1 shows the distribution of activities during the 8 time slots performed by the elderly subject in the first 10 days. The volunteers received an emergency alert during the ninth day from the subject’s device, which is also evident by looking at the 09-12 time slot in Figure 1, where most of the activity values lie above the mid-way mark, and only some (actually 1) falls. On inquiring about the subject, we were informed that the subject felt uneasy and tired, and nothing serious was there. Thus, this unusual activity performing pattern of the subject resulted in a true alarm being generated. We observed several false alarms in the first few days, but the system started demonstrating gradual improvement in performance after the early 10 days of learning was over.

**FIGURE 1:**
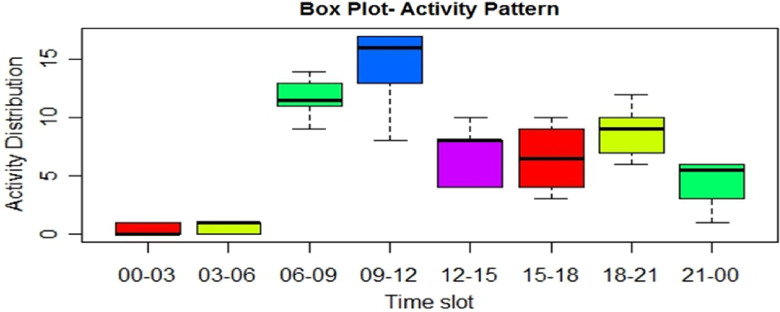
Box Plot Activity Pattern After 10 Days of Monitoring.

Based on the intimation alarms generated in the first 60 days of the pilot study, as shown in Table 2, the authors observed that the number of false positives (ie, the report that the subject was sick when actually healthy and active) totaled 65, and the number of false negatives (ie, the subject was reported as healthy when actually sick) totaled 31. The number of true positives (ie, the subject was reported as sick when actually sick) was observed as 14. The number of true negatives (ie, the subject was reported as healthy when actually healthy) was recorded as 345. Thus, the accuracy of the system was found to be 78.89%.

**TABLE 2:** Observations for the First 60 Days

Observations Based on the First 60 Days and 455 Time Slots		True Condition	
Predicted Condition		Sick	Healthy
	Sick	14	65
	Healthy	31	345

## CONCLUSION

During the COVID-19 outbreak, older people who are living alone are often the most vulnerable. When the pan-region lockdown is in force, people are not even visiting their neighbors. In such conditions, taking care of the elderly is of utmost importance. This study reports on the efforts the authors made with ensuring the well-being of 1 elderly research subject. The proposed system provides a comprehensive solution for elder care during disastrous circumstances. The system not only offers the facility for home delivery of the necessary items on demand to the elderly, but also ensures that proper help reaches the individual during critical times. The proposed system provided 24/7 vigilance of the elderly subject without the need for any video surveillance; thus, the system respected the privacy of the elderly individual.

The reported system didn’t require the research subject to wear any gadget or band on his wrist, which some people find cumbersome. The major bottleneck in implementing the presented system lies in the unavailability of health care data as an electronic health record, which is still not on the agenda in many developing countries. As a future course of action, the presented study can be extended by considering multiple subjects with better conditions post-COVID-19.
